# Biodegradable Electrospun Nonwovens Releasing Propolis as a Promising Dressing Material for Burn Wound Treatment

**DOI:** 10.3390/pharmaceutics12090883

**Published:** 2020-09-17

**Authors:** Mateusz Stojko, Jakub Włodarczyk, Michał Sobota, Paulina Karpeta-Jarząbek, Małgorzata Pastusiak, Henryk Janeczek, Piotr Dobrzyński, Gabriela Starczynowska, Arkadiusz Orchel, Jerzy Stojko, Olgierd Batoryna, Paweł Olczyk, Katarzyna Komosińska-Vassev, Krystyna Olczyk, Janusz Kasperczyk

**Affiliations:** 1 Centre of Polymer and Carbon Materials, Polish Academy of Sciences, M. Curie-Skłodowskiej 34, 41-819 Zabrze, Poland; jwlodarczyk@cmpw-pan.edu.pl (J.W.); msobota@cmpw-pan.edu.pl (M.S.); pkarpeta@cmpw-pan.edu.pl (P.K.-J.); mpastusiak@cmpw-pan.edu.pl (M.P.); henryk.janeczek@cmpw-pan.edu.pl (H.J.); pdobrzynski@cmpw-pan.edu.pl (P.D.); jkasperczyk@cmpw-pan.edu.pl (J.K.); 2 Department of Biopharmacy, Faculty of Pharmaceutical Sciences in Sosnowiec, Medical University of Silesia in Katowice, Jedności 8, 41-200 Sosnowiec, Poland; gabriela.starczynowska@gmail.com (G.S.); aorchel@sum.edu.pl (A.O.); 3 Department of Toxicology and Bioanalysis, Faculty of Pharmaceutical Sciences in Sosnowiec, Medical University of Silesia in Katowice, Ostrogórska 30, 41-200 Sosnowiec, Poland; jstojko@sum.edu.pl; 4 Department of Community Pharmacy, Faculty of Pharmaceutical Sciences in Sosnowiec, Medical University of Silesia in Katowice, Kasztanowa 2, 41-205 Sosnowiec, Poland; obatoryna@sum.edu.pl (O.B.); polczyk@sum.edu.pl (P.O.); 5 Department of Clinical Chemistry and Laboratory Diagnostics, Faculty of Pharmaceutical Sciences in Sosnowiec, Medical University of Silesia in Katowice, Jedności 8, 41-200 Sosnowiec, Poland; kvassev@sum.edu.pl (K.K.-V.); olczyk@sum.edu.pl (K.O.)

**Keywords:** electrospinning, drug delivery, PLGA, propolis, burn wounds

## Abstract

The selection of dressing is crucial for the wound healing process. Traditional dressings protect against contamination and mechanical damage of an injured tissue. Alternatives for standard dressings are regenerating systems containing a polymer with an incorporated active compound. The aim of this research was to obtain a biodegradable wound dressing releasing propolis in a controlled manner throughout the healing process. Dressings were obtained by electrospinning a poly(lactide-*co*-glycolide) copolymer (PLGA) and propolis solution. The experiment consisted of in vitro drug release studies and in vivo macroscopic treatment evaluation. In in vitro studies released active compounds, the morphology of nonwovens, chemical composition changes of polymeric material during degradation process, weight loss and water absorption were determined. For in vivo research, four domestic pigs, were used. The 21-day experiment consisted of observation of healing third-degree burn wounds supplied with PLGA 85/15 nonwovens without active compound, with 5 wt % and 10 wt % of propolis, and wounds rinsed with NaCl. The in vitro experiment showed that controlling the molar ratio of lactidyl to glycolidyl units in the PLGA copolymer gives the opportunity to change the release profile of propolis from the nonwoven. The in vivo research showed that PLGA nonwovens with propolis may be a promising dressing material in the treatment of severe burn wounds.

## 1. Introduction

Burn wounds are a very important therapeutic problem, causing a significant deterioration of patients’ life quality. Wound healing can be a long-term process, often associated with troublesome infections, pain and unaesthetic scarring. Due to this, modern methods of treatment are sought to accelerate the repair of tissues as much as possible and minimize or eliminate complications [1,2,3]. The most common forms of drugs currently used are: solutions, suspensions, creams, emulsions and ointments [4]. The most popular active substances in the treatment of hard-to-heal wounds are iodopovidone, silver ions (e.g., in form silver sulfadiazine, sulfasalazine, silver nitrate), polyhexamethylene, biguanide, octenidine, phenoxyethanol and chlorhexidine, as well as antibiotics [4,5,6,7,8]. These substances are effective in the initial stages of healing, but the problem related to their use is the short duration of activity at the damaged tissue area. Especially in the case of wounds characterized by high exudation, these preparations become too mobile [2,9,10]. Materials used traditionally for dressings, such as gauze, plaster or bandage, are mainly intended to protect the wound from contamination and its mechanical protection. Modern dressings are designed to not only cover the surface of the wound, but also exert a beneficial effect on the healing process and minimize complications [11]. Effective healing is possible due to the appropriate choice of dressing, which will ensure optimal conditions for the proper course of the healing process. The ideal dressing should provide a moist wound environment, accelerate re-epithelialization, accelerate angiogenesis and synthesis of connective tissue, allow gas exchange between the wound and the environment, provide optimal wound temperature to increase blood flow within it, pose barrier to infection, not adhere to the wound, minimize unpleasant smell, support the migration of leukocytes and enzymes, be transparent, allow observation of healing process, be sterile, be non-toxic and be non-allergic [11]. Among modern dressing materials substances are distinguished as: alginates, collagens, chitosan and other polymeric materials in form of foams, hydrogels and films [2,3,12]. Interesting alternatives for standard polymeric dressings are complex dressings containing incorporated active compounds [13]. Recently, polymeric dressings in the form of nonwovens obtained by the electrospinning process have caught the attention of researchers. Electrospinning is a processing technique where fibers are formed from molten polymers or polymer solutions using a generated electric field. By using this technique, it is possible to control the pharmacokinetic processes in such a way as to obtain a prolonged action of the active ingredient within the wound healing. These types of dressings are characterized by a number of beneficial properties, such as a large surface area and porous structure, better gas exchange and more effective absorption of exudate, protection against infections and dehydration, as well as the ease of introducing the active substance [1,2,3,14,15,16].

As drug carrier polymers, poly(lactide) (PLA), poly(lactide-*co*-glycolide) (PLGA) and poly(ε-caprolactone) (PCL) are most commonly used [14,17]. A lot of research has shown that PLGA copolymer is a biodegradable, biocompatible, non-toxic and non-immunogenic material that can be used as a carrier for a controlled release of drugs [18,19]. Therefore, it can be used in various fields of medicine, including being used as a dressing material in the treatment of wounds [20]. By using components with antibacterial properties, such as silver nanoparticles, antibiotics, antioxidants or natural substances, one of the biggest challenges facing modern dressings is possible to achieve: ensuring a sterile wound environment and other favorable conditions for its treatment [21].

Different substances having specific therapeutic properties can be incorporated into electrospun fibers. One of the natural substances of bee origin used in medicine for centuries, which can be incorporated into polymer fibers, is propolis. This apitherapeutic is a sticky, plant-based resin substance, formed from resins to which wax and secretions of the throat and mandibular glands of bees are added. The chemical composition of propolis is varied, about 300 components of this apitherapeutic have been discovered and identified thus far [22,23]. Propolis has wide range of therapeutic properties, such as antibacterial, antifungal, antiviral, antioxidant, anti-inflammatory, immunostimulatory and antineoplastic effects. What is most important is that it stimulates the regeneration of wounds very well [24,25,26,27,28,29]. Recently, propolis has aroused great interest due to its many properties, and thus multifaceted operation. Its antibacterial properties, beneficial for the wound healing process, play a fundamental role in many papers [30,31,32,33]. This was confirmed in studies conducted by Kabała-Dzik et al. [34], who showed that propolis balm compared to silver sulfadiazine, which is frequently used in the treatment of skin burns, gives better therapeutic results, which was manifested by a significant acceleration of recovery process and bacteriostatic activity of apitherapeutic substance in relation to *Staphylococcus aureus* and germicidal activity in relation to *Bacillus* spp., *Enterococcus faecalis* and *Candida albicans*. Propolis also has anti-inflammatory, antioxidant and regenerative effects of wound healing and prevention of scarring [30]. The beneficial effect of propolis on experimental wounds was verified by series of studies carried out by Olczyk et al. [26,27,28,29] concerning biochemical analyses, which proved the therapeutic effectiveness of bee products in the process of repairing tissue damage.

Electron paramagnetic resonance (EPR) spectroscopic examination of different types of paramagnetic centers in the blood during healing of skin burned wounds revealed that innovative PLGA nonwoven dressings strongly influence the oxidative-antioxidative balance during the burn wound healing process [35]. In relation with the above, in many experiments on the production of polymer fibers by electrospinning, propolis is used as a therapeutic substance [14,32,36,37].

The aim of this study was to obtain a nonwoven dressing made of a biocompatible and biodegradable polymer, for the treatment of burn wounds, which releases propolis in a controlled manner throughout the healing process. Additionally, the goal was to develop a material with features that can ensure favorable conditions for regeneration of damaged tissues, sterile wound environment and, finally, degradation after the end of the treatment, allowing to avoid discomfort associated with removal of the dressing. The development of a series of dressings with different release profiles and degradation times would allow the application of appropriate dressing to the type and severity of the wound, and thus, is relevant for usage in personalized wound treatment. 

## 2. Materials and Methods

### 2.1. Monomers and Initiator

Monomers: l-lactide and glycolide (Foryou Medical Devices Co., Ltd., Huizhou, China) were purified by recrystallization from anhydrous ethyl acetate and then dried in a vacuum oven at room temperature until constant weight was obtained. Initiator: zirconium (IV) acetylacetonate; Zr(acac)_4_, (Sigma-Aldrich, Merck KGaA, Darmstadt, Germany) was used as received. 

### 2.2. Copolymerization Procedure

A series of poly(l-lactide-*co*-glycolide) with various compositions (Table 1) were synthesized according to the method described in the literature [38] in bulk via the ring opening polymerization (ROP) of l-lactide and glycolide. A typical copolymerization was as follows. Weighed amounts of l-lactide, glycolide and zirconium (IV) acetylacetonate (Zr(acac)_4_) (initiator/comonomers ratio of 1:600) were charged into dried, a two-necked glass flask. Then, the flask was degassed under vacuum for 5 min, refilled with dry argon and sealed. Next, the reaction vessel was conditioned on an oil bath equipped with a periodically working shaker at 130 °C for 24 h and then at 115 °C for 72 h. The copolymers thus obtained were purified by dissolution in chloroform (Avantor Performance Materials Poland S.A., Gliwice, Poland) and precipitation into cold methanol (Avantor Performance Materials Poland S.A., Gliwice, Poland) in order to remove the unreacted monomers, followed by drying under a vacuum at room temperature to constant weight.

### 2.3. Preparation of Polymer Nonwovens by Electrospinning

Samples without active compound: the copolymers PLGA 50/50 (18% *w*/*w*), PLGA 70/30 (15% *w*/*w*) and PLGA 85/15 (18% *w*/*w*) were dissolved in the mixture of solvents: chloroform (Sigma Aldrich) and 1,1,1,3,3,3-hexafluoro-2-propanol (HFIP) (Sigma Aldrich, Merck KGaA, Darmstadt, Germany) in a volume ratio of 4:1 (*v*/*v*). Samples containing propolis: the copolymers PLGA 50/50 (18% *w*/*w*), PLGA 70/30 (15% *w*/*w*) and PLGA 85/15 (18% *w*/*w*) were dissolved in chloroform and mixed with propolis stock solution (0.5 g/mL in HFIP (*w*/*w*) prepared from dry propolis extract (Apipol-Farma, Myślenice, Poland) to obtain 5% (*w*/*w*) and 10% (*w*/*w*) propolis concentration relative to the amount of polymer. The mixture was topped up with an amount of HFIP so that its ratio to chloroform was 1:4 (*v*/*v*). The procedure of preparing solutions was repeated analogously for all tested polymers. 

In the next step, solutions were used for obtaining nonwovens with TL-Pro-BM electrospinning unit (Tong Li Tech, Shenzhen, China). The device was equipped with two high voltage power supplies. First, for generating a positive electrical potential, a potential of 21 kV was applied to the spinneret, in the form of a G20 steel needle. The second one was applying a negative potential of −7 kV to the fiber collector, in form of a steel mandrel of 27 mm diameter, rotating at a rate of 400 RPM. Distance between electrodes was set to 21 cm. Polymer solutions was dosed to the spinning nozzle through a capillary at 3 mL/h, by using Harvard Apparatus PHD Ultra 4400 (Harvard Apparatus, Cambridge, MA, USA) syringe pump. The average dosing volume was 23 ± 2 mL. The temperature inside the chamber during electrospinning was 17 ± 0.2 °C, while the relative humidity was changing between 29% and 42%. Nonwovens were obtained in form of 27 cm × 8.5 cm sheets. The procedure was repeated analogously for all solutions. Obtained nonwovens were dried under a vacuum at room temperature to constant weight.

### 2.4. In Vitro Degradation

In vitro degradation study of nonwovens obtained by the electrospinning process was carried out in 5 mL of 0.01 M phosphate buffered saline water solutions (PBS, pH 7.4) at 37 °C for 84 days. 10 mm × 5 mm samples were cut out, from each kind of the mats.

Buffer sampling for drug release measurement and nonwovens sampling for determination of degradation rate were done at predefined time intervals. The degradation rate was characterized by changes in copolymer composition. Additionally, weight loss and water absorption were measured and Surface Electron Microscopy (SEM) pictures were made, to assess visual changes in surface morphology during the experiment. 

### 2.5. In Vitro Drug Release

Drug release was realized under in vitro conditions at 37 °C in PBS (pH 7.4) for 84 days. After sampling at the predetermined intervals buffer was replaced. The samples were collected for quantitative analysis using UV–VIS spectrometry to measure the amount of released drug (Spectrophotometer Spark 10M, TECAN, Männedorf, Switzerland). Analysis were performed at the wavelength of 337 nm [39]. The relationship between absorbance and propolis concentration was determined on the basis of a calibration curve.

### 2.6. Characterizations and Analysis

Nonwovens were analyzed before, during and after degradation process by the methods described in the following paragraphs.

The morphology of nonwovens was analyzed by Surface Electron Microscope (Quanta 250 FEG, FEI Company, Hillsboro, OR, USA) operating in low vacuum conditions (80 Pa), with an acceleration voltage of 5 kV, from secondary electrons collected by Large Field Detector(FEI Company). Average diameters of fibers were calculated using ImageJ software.

Copolymers composition changes were determined using ^1^H nuclear magnetic resonance (Avance II Ultrashield Plus 600 MHz, Bruker, Billerica, MA, USA) (NMR) on every stage of degradation.CDCl_3_ was used as a deuterated solvent. The ^1^H NMR spectra were obtained at 22 °C with 64 scans, 1 s acquisition time and 11 µs pulse. Additionally, weight loss and water absorption were calculated.

Thermal properties were determined using DSC Q2000 Differential Scanning Calorimeter (DSC) (TA Instruments, New Castle, DE, USA). The specimens were heated from −30 to 200 °C under a nitrogen atmosphere (flow 50 mL/min) at a heating rate of 20 °C/min. The glass transition temperature (T_g_) was determined as the midpoint of heat capacity change of the amorphous sample obtained by quenching the melt by using liquid nitrogen.

After the synthesis molar masses were determined. The molar mass and the polydispersity index of the copolymers were determined by means of gel permeation chromatography (GPC). The experiments were conducted in THF solution at 35 °C (flow rate 1 mL/min) using a Spectra-Physics SP 8800 gel permeation chromatograph (Spectra-Physics, Santa Clara, CA, USA) equipped with IR detector Shodex SE 61 (Shodex, Tokyo, Japan). The column configuration consisted of two stryragel-packed columns 500 Å (Polymer Laboratories Ltd., Church Stretton, UK). The results were calculated based on the polystyrene calibration curve.

### 2.7. In Vivo Assessment 

**Scheme 1:**
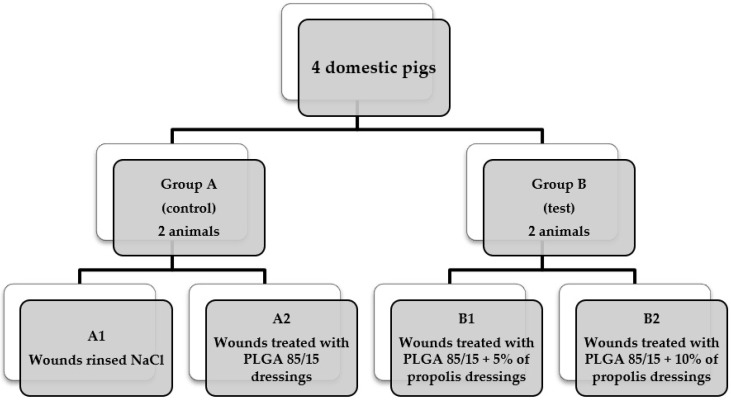
Research groups for in vivo study.

For the in vivo study, nonwovens were made according to procedures described above from PLGA 85/15 polymer. Four domestic pigs, weighting about 50 kg and with an age of 16 weeks, were used. Both in the adaptive and experimental period, they were in standard zoohygienic conditions and fed a full blend of feed. The 21-day experiment consisted in observation of healing burn wounds supplied with tested nonwovens. Burn wounds (1.5 cm × 3 cm) were obtained by applying an electrode heated to a constant temperature 170 °C for 10 s, with a constant pressure of 270 g. Research was made according to the assumptions of the standard Hoekstra model, in accordance with the Dutch Law of Animal Research and the experimental protocol of the Charity Committee at the University of Amsterdam, with the consent of the Ethics Committee of the Medical University of Silesia (LKE-111/2014, date of approval: 24.11.2014). All invasive procedures were performed under general anesthesia. Four research groups were set according to the scheme below (Scheme 1).

Healing processes were compared based on the macroscopic changes observed after 3, 5, 10, 15 and 21 days of treatment.

## 3. Results

### 3.1. In Vitro Degradation

The study of the change in the thermal properties of materials related to different content of active compound by the differential scanning calorimetry (DSC) (Table 2) showed that the glass transition temperature (T_g_) decreases with increasing propolis content in the sample. It seems that the active substance introduced into the polymer is, to some extent, compatible with the matrix and causes plasticization of the material.

**Figure 1:**
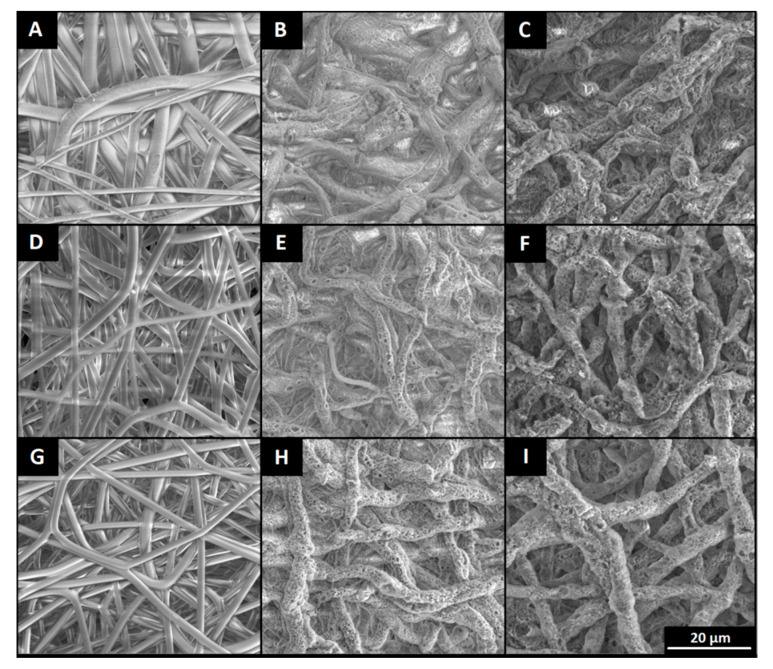
SEM images of the nonwoven materials obtained by electrospinning (5000×); (**A**–**C**),-poly(l-lactide-*co*-glycolide) 50/50—before and after 6 and 12 weeks of degradation, respectively; (**D**–**F**),-poly(l-lactide-*co*-glycolide) 50/50 + 5% of propolis—before and after 6 and 12 weeks of degradation, respectively; (**G**–**I**) poly(l-lactide-*co*-glycolide) 50/50 + 10% of propolis—before and after 6 and 12 weeks of degradation, respectively.

**Figure 2:**
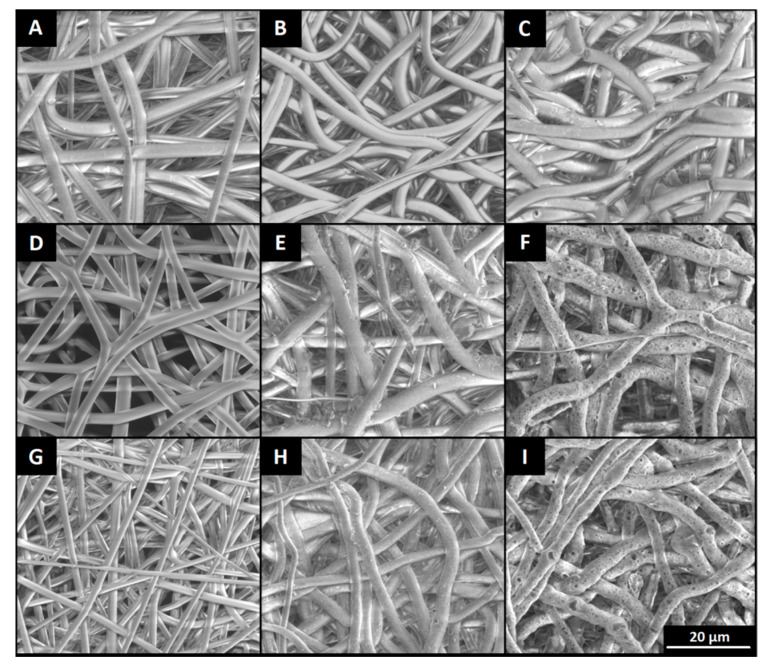
SEM images of the nonwoven materials obtained by electrospinning (5000×); (**A**–**C**)-poly(l-lactide-*co*-glycolide) 70/30—before and after 6 and 12 weeks of degradation, respectively; (**D**–**F**),-poly(l-lactide-*co*-glycolide) 70/30 + 5% of propolis—before and after 6 and 12 weeks of degradation, respectively; (**G**–**I**)-poly(l-lactide-*co*-glycolide) 70/30 + 10% of propolis—before and after 6 and 12 weeks of degradation, respectively.

**Figure 3:**
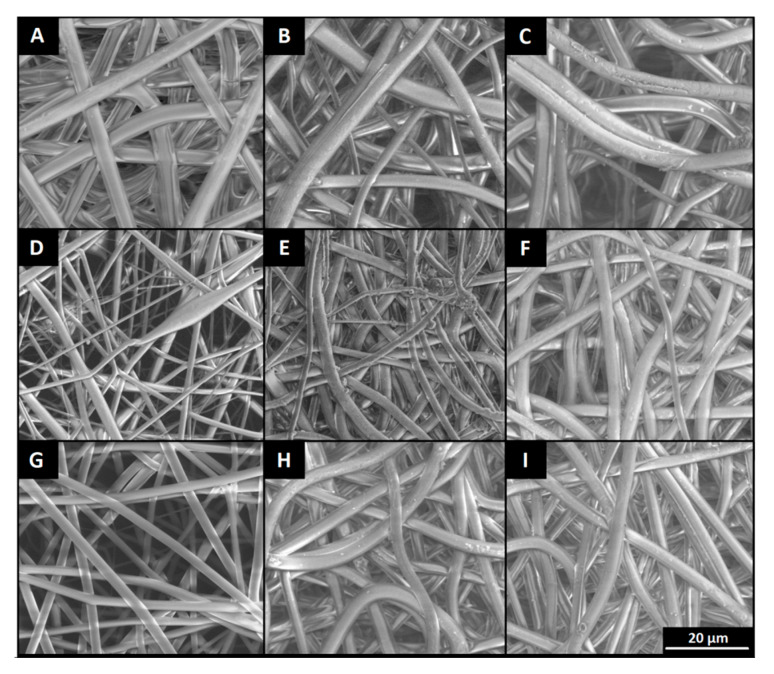
SEM images of the nonwoven materials obtained by electrospinning (5000×); (**A**–**C**)-poly(l-lactide-*co*-glycolide) 85/15—before and after 6 and 12 weeks of degradation, respectively; (**D**–**F**),-poly(l-lactide-*co*-glycolide) 85/15 + 5% of propolis—before and after 6 and 12 weeks of degradation, respectively; (**G**–**I**)-poly(l-lactide-*co*-glycolide) 85/15 + 10% of propolis—before and after 6 and 12 weeks of degradation, respectively.

SEM analysis revealed that fibers before the experiment were characterized by a regular, elongated, smooth structure. They were arranged chaotically and did not have visible deformation. Only PLGA 85/15 with 5 wt % addition of propolis had a single bead. During degradation, PLGA 50/50 fibers without active compound and with active compound in both concentrations lost their initial shape and integrity before the sixth week of degradation (Figure 1). PLGA 70/30 fibers with addition of bee product in both concentrations has become porous around 12 weeks of degradation (Figure 2). PLGA 85/15 fibers have kept their initial shape throughout the whole period of degradation (Figure 3).

There is a clear relationship between the mutual ratio of comonomers and the damage of fibers during degradation. A higher content of glycolidyl units in the initial composition causes faster formation of pores, leading to loss of the fibrous structure.

**Figure 4:**
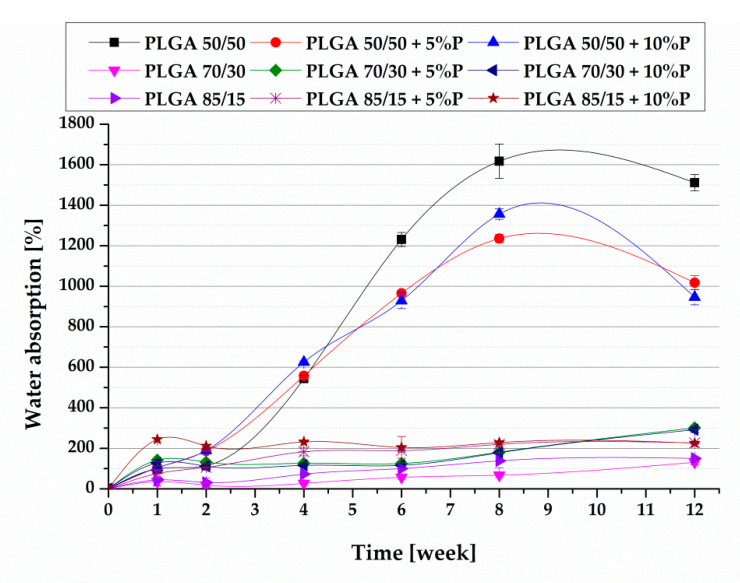
Percentage of water uptake of the nonwovens during the 12-week incubation ($\bar{x}\pm SD,\;N=3$).

**Figure 5:**
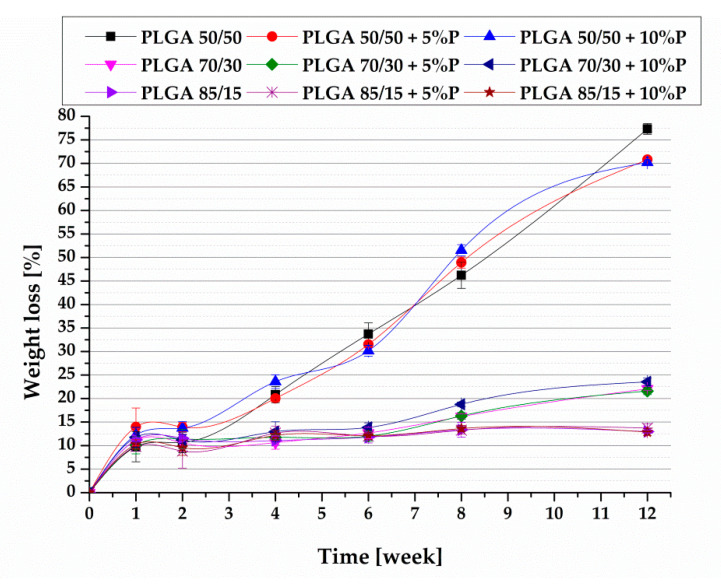
Percentage of weight loss of the nonwovens during the 12-weeks of incubation ($\bar{x}\pm SD,\;N=3$).

Weight loss and water uptake results are presented on the Figure 4 and Figure 5.

Based on Figure 4 presented above, it can be concluded that the highest water absorption level, (about 1000–1600%) showed nonwovens with a 50/50 mutual comonomer ratio around the eight week of incubation. Then, water absorption was lower, which is caused by the highly advanced fibrous structure damage. The lowest water absorption was observed for nonwovens with molar ratios of 70/30 and 85/15 without active compounds. Samples without propolis, in the case of PLGA 70/30 and 85/15 polymer, were characterized by lower water absorption than their counterparts with active substances. The exceptions were 50/50 nonwovens without hydrophobic apitherapeutic, which showed the highest water absorption due to the least hydrophobic character of all materials tested.

The obtained results (Figure 5) indicate that the largest weight loss occurred in PLGA 50/50 samples, and it was about 77% of the initial weight of samples without active compounds and 70% of the initial weight for both samples with the addition of bee product. The difference can be caused by the presence of a hydrophobic active compound, which decreases the penetration of water through fibers and slows down the weight loss slightly. In PLGA 70/30 and PLGA 85/15 samples, weight loss after 12 weeks of incubation was much lower. Fast weight loss was observed only in the first week, which can be caused by rinsing the low molecular fraction of polymer. In case of 70/30 samples, weight loss after 12 weeks was about 22–23% of initial weight and slightly increased after the sixth week, which may be due to the reduction in quantity of hydrophobic active substance inside fibers; hence, fibers became slightly more hydrophilic. Eluting bee product from fibers also causes formation of pores, which was also observed on SEM pictures, and allows the incubation medium to penetrate fibers easier; hence, weight loss is faster. Weight loss of PLGA 85/15 samples after 12 weeks of degradation was about 13–14% and was stable after the first week of incubation.

**Figure 6:**
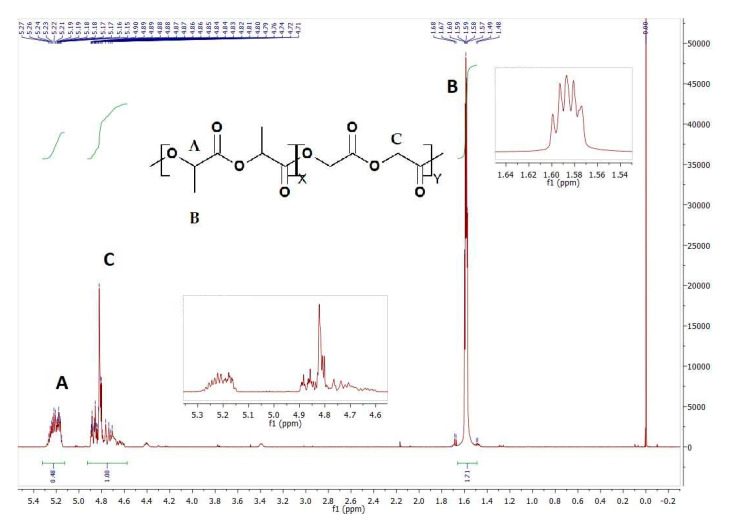
^1^H NMR spectra of electrospun PLGA 50/50 nonwovens (**A**): –CH range of lactidyl units, (**B**): –CH_3_ range of lactidyl units, (**C**): –CH_2_ range of glycolidyl units.

**Figure 7:**
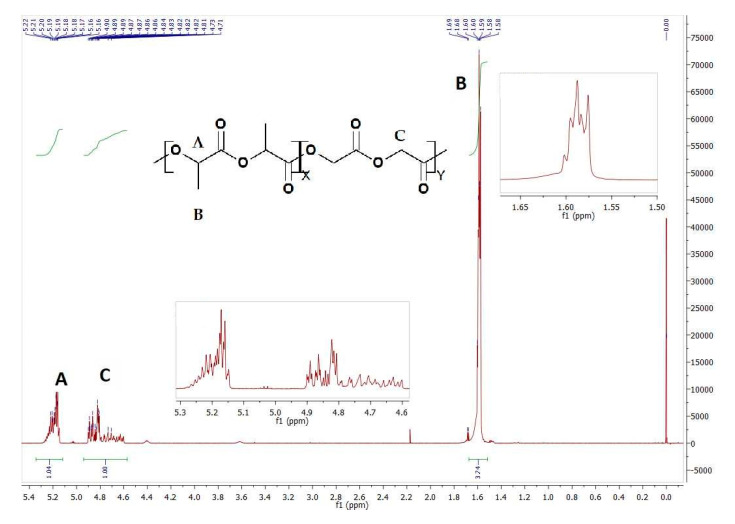
^1^H NMR spectra of electrospun PLGA 70/30 nonwovens (**A**): –CH range of lactidyl units, (**B**): –CH3 range of lactidyl units, (**C**): –CH2 range of glycolidyl units.

**Figure 8:**
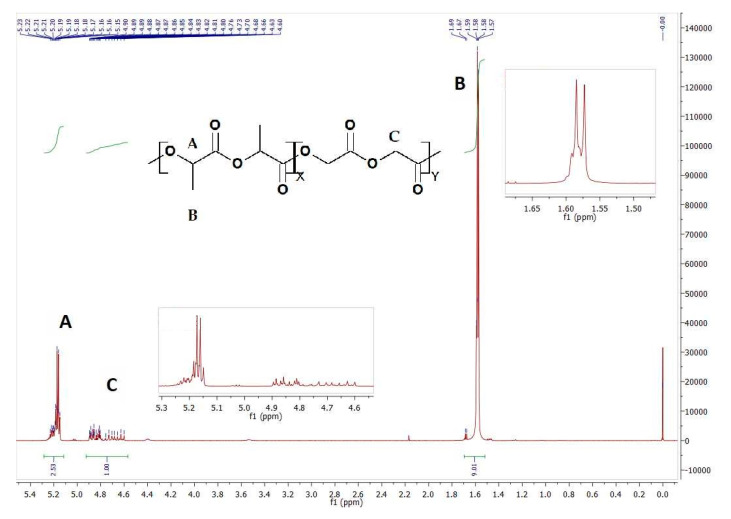
^1^H NMR spectra of electrospun PLGA 85/15 nonwovens (**A**): –CH range of lactidyl units, (**B**): –CH_3_ range of lactidyl units, (**C**): –CH_2_ range of glycolidyl units.

To determine the changes in polymer composition during degradation, ^1^H NMR spectra were taken. The spectra of polymers at the beginning of experiment (Figure 6, Figure 7 and Figure 8) and the results of the analysis of copolymer composition changes (Table 3) are presented below.

The unit ratio of PLGA 85/15 samples after 12 weeks of hydrolytic degradation remained almost unchanged (Table 3). No change in the molar composition of the copolyester indicates poor polymer degradation. Analyzing the composition changes of the PLGA 70/30 material, for each type of sample at a given measuring point, the unit ratio was almost the same. In the case of the most hydrophilic of the tested materials—PLGA 50/50—the effect of the addition of propolis is complex. Rapid diffusion of water into fibers caused the unit ratio changes to be the largest among all materials tested. In the initial stage (up to six weeks) of the experiment, the comonomeric unit content changes were almost the same for all 50/50 samples. In the case of samples containing 5% and 10% of propolis, the diffusion processes mentioned were probably disturbed—after 12 weeks of degradation, changes in molar composition were lower than in materials without active substances, which may be due to the presence of a hydrophobic active substance.

**Figure 9:**
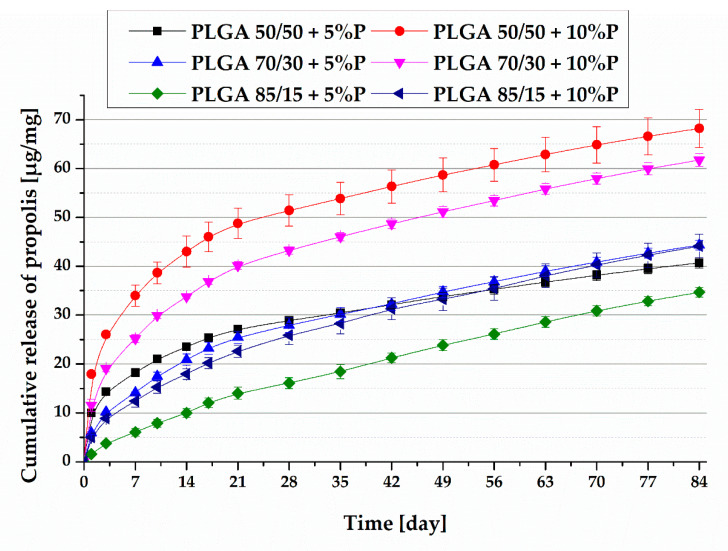
Cumulative release of propolis from biodegradable nonwoven materials (PLGA 50/50, PLGA 70/30, PLGA 85/15) obtained by electrospinning expressed by micrograms of released active compound per 1 milligram of nonwoven material ($\bar{x}\pm SD,\;N=3$).

Based on the results presented above, it can be stated that the higher the initial content of the more hydrophilic glycolidyl units, the faster their content in the polymer decreases, and thus, the faster degradation of such a material. According to the assumptions, the fastest decrease in the content of glycolidyl units was observed in samples 50/50, slower in samples 70/30, while the composition of 85/15 nonwovens did not significantly change during the 12-week incubation—this resulted in the slowest release of propolis among all the materials tested (Figure 9). The results obtained on the basis of NMR analysis are reflected in the other presented results.

### 3.2. Drug Release

Presented data showed that the release of the active compound was the highest for PLGA 50/50 nonwovens, and the lowest for PLGA 85/15 nonwovens. The same relationship was observed in case of burst effect. It was stronger in the case of a polymer with 50% initial content of GG units (especially samples with 10% content of propolis) and low in the case of nonwoven mats, with the lowest initial content of this hydrophilic comonomer. PLGA 85/15 nonwovens with a 5% content of apitherapeutic were characterized by drug release kinetics closest to the zero order kinetics. From the presented results, it can be concluded that release was strongly dependent on propolis content in the polymer carrier. At individual time points, from PLGA 50/50 mats with 10% of active compound, almost twice as much drug was released than from PLGA 50/50 with 5% content of propolis. This is due to the two-fold higher concentration of the active substance in the first matrix and the dynamic degradation caused by the high initial content of glycolidyl units, which degrades faster than the lactidyl units. In the case of mats with compositions 70/30 and 85/15 with 10% addition of propolis, no comparable amount of released drug was observed relative to the mat with 5% addition, but it was also significantly higher.

The observed ability to control the propolis release profile allows, to a large extent, to adjust the type of the formed dressing to the requirements of the selected therapy. However, studies to date do not indicate which release profile is optimal for treating deep burns. The wound healing process depends on the degree and depth of the tissue damage. In case of a model burn wound, a constant amount of released substance is beneficial in all phases of repair processes. Even after the epithelialization process is finished, a constant concentration of active substance allows the scar maturation processes to be accelerated. A well-known phenomenon is the influence of propolis on the increase of concentration of glycosaminoglycans, which are directly responsible for all stages of repair processes. For this reason, for in vivo tests, we selected the nonwoven matrix (based on PLGA 85/15) that allows the release of propolis over a long time with an approximately constant released dose.

### 3.3. In Vivo Assessment

For the in vivo test, nonwovens were produced from PLGA 85/15 (l-LA:GL ratio: 82:18, M_n_ = 60 kDa, M_w_ = 156 kDa) polymer. The material was chosen because of its well characterized processes of degradation and release of the active substance in vitro. Preliminary results are presented solely for the initial confirmation of the potential therapeutic efficacy of the obtained nonwovens. In order to obtain more accurate results, further tests will be carried out on samples taken during the experiment.

**Figure 10:**
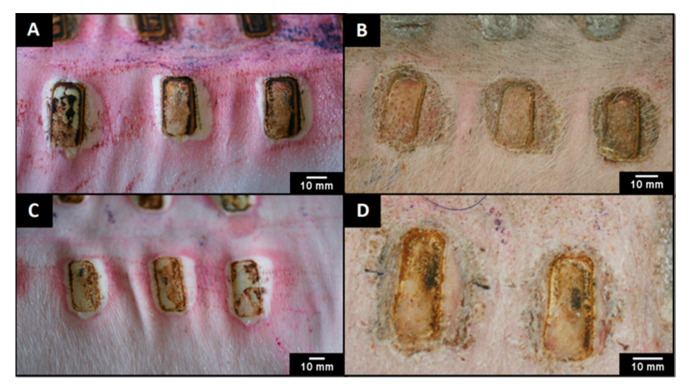
Pictures of burn wounds: rinsed twice a day with NaCl before (**A**) and after 21 days of treatment (**B**); treated with PLGA 85/15 dressings before (**C**) and after 21 days of treatment (**D**).

**Figure 11:**
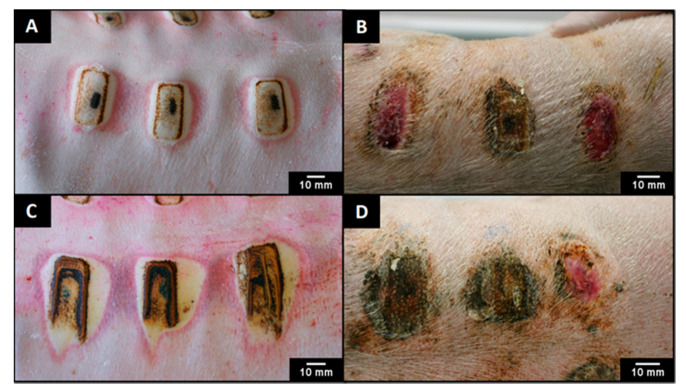
Pictures of burn wounds: treated with PLGA 85/15 with 5% of propolis dressings before (**A**) and after 21 days of treatment (**B**); treated with PLGA 85/15 with 10% of propolis dressings before (**C**) and after 21 days of treatment (**D**).

The macroscopic image of the burn wounds (Figure 10A,C and Figure 11A,C) before treatment can be described as follows: necrosis at the place of burn induction and up to 3–15 mm from its edge, intensive reddening and edema around the necrotic area. The exudation and carbonization of tissue was also observed. After 21 days of treatment, wounds treated with individual methods clearly differed from each other. Wounds rinsed with NaCl twice a day (control of unsupported healing process) (Figure 10B) were covered by a thick scab. The area of burn induction was surrounded by edema. Wounds treated with PLGA nonwovens without active compounds (Figure 10D) were covered by flexible, thin scabs, peeling off on the wound edges. Places of burn induction were marked, and there was no edema. Wounds treated with dressings with 5% of propolis (Figure 11B) were covered with epidermis and their area reduced. There were small fragments of flexible, peeling scabs, and growing bristles were visible. Edema and inflammation did not occur. Wounds treated with nonwovens with 10% of apitherapeutic (Figure 11D) were covered with epidermis, and their area was reduced, some fragments of scabs left. At the site of burn induction area, the growing bristles were visible. Edema and inflammation did not occur. The best therapeutic effect based on macroscopic evaluation was observed in the case of dressings with 5% of propolis, because healing processes of wounds supplied with these nonwovens was highly advanced—they were characterized by the smallest area, the thinnest scab and most advanced regrown of epidermis and bristles. Furthermore, the appearance of epidermis and bristles means no signs of scar formation, which indicates a lack of unaesthetic consequences of the healing processes. 

## 4. Discussion

Due to the fact that propolis is characterized by the pluripotent action enhancing the treatment of skin and tissue damages, it seemed advisable to carefully examine its release profile from polymeric nonwoven obtained by the electrospinning process [22,24,25]. It was also reasonable to conduct an experiment using a PLGA mat with different lactidyl units to glycolidyl units ratio, as confirmed by many studies; the effect of the unit amount change in copolymer on release of active substances gives the opportunity to match it in such a way to achieve the expected effect of treatment of specific types of wound [18,20,40]. Furthermore, these nonwovens are non-toxic due to their biodegradability and biocompatibility, and the fact that they provide adequate conditions for a proper healing process makes them good carriers for active compound [18,19].

Selection of an appropriate ratio of lactidyl to glycolidyl units in the copolymer determines its degradation rate and the kinetics of drug release from polymer nonwoven [20]. Results indicate that the largest weight loss and water absorption after 12 weeks of incubation were PLGA 50/50 mats. However, the smallest weight loss and water absorption was found in the case of PLGA 85/15 and 70/30 nonwovens. On the other hand, the lowest water absorption was found in PLGA 70/30 and 85/15 dressings without the addition of apitherapeutic. It means that samples that have a relatively higher amount of more hydrophilic glycolidyl units in their composition degraded most rapidly. In the research conducted by Blackwood et al. [41] on the samples of the same copolymer with three different lactidyl to glycolidyl units ratio (50/50, 75/25 and 85/15), the same relationship was proven. Furthermore, Zong et al. [40] investigated PLGA 10/90 copolymer degradation. It was shown that in the short period of time (about 16 days), the polymer matrix disintegrated, which confirms the relationship described in our research. The hydrophilic degradation affects the release rate of drug from the matrix; the faster the degradation occurs, the faster the drug will be released. The results of our work show that the fastest and the largest amount of propolis was released from PLGA 50/50 samples. In turn, the lowest release rate and amount of drug was released from the PLGA 85/15 samples, which coincides with the degradation results. It was noted that at selected time points, PLGA 50/50 with 10% content of drug samples released two times more propolis than PGLA 50/50 samples with 5% content. However, for the remaining copolymers, the analogous differences are not so large, which means that for these carriers the composition and structure of the mat had a greater impact on the release profile of the drug. The difference lies in the character of polymer that is used to make the nonwovens. Studies performed by Kim et al. [36], Adomavičūtė et al. [14] and Sutjarittangtham et al. [37] proved that the type of polymers from which the dressing are made is of key importance in changing the release profile of the drug. Results of their studies also confirmed that propolis is released slower from the PLA and PU nonwoven than from polymers with greater hydrophobicity. In these experiments, the antibacterial properties of mats with propolis were also confirmed, which proved that it could be successfully used as wound dressing. 

Preliminary results of in vivo studies reflect the state of knowledge arising from other studies on wound healing with apiculture products [30,33], especially studies carried out on a similar experimental model that have proven the beneficial effect of propolis on the treatment of skin and tissue damage by biochemical methods [26,27,28,29]. The macroscopic evaluation of wound healing showed that propolis released from the nonwoven promotes burn wound healing. Furthermore, the advantage of obtained dressings is that there is no need for daily supply of the active substance in the form of an ointment or cream, or to change dressing such as gauze. 

Moreover our recent results of laboratory examinations, of blood samples taken from the experimental animal, regarding the regulatory properties of the mentioned, innovative dressing—strongly influencing the oxidative-antioxidative balance—indicate among others that during the initial phase of burn wound healing, the highest amount of paramagnetic centers in the form of the high-spin Fe^3+^ in methemoglobin, and, respectively, the lower amounts of the high-spin Fe^3+^ in transferrin were observed; the amount of the high-spin Fe^3+^ in methemoglobin blood decreased after 21 days of tissue repair; the amount of the high-spin Fe^3+^ in transferrin moderately increased after 21 days of wound management with the use of biodegradable dressing [35]. Furthermore, a favorable effect of innovative biodegradable apitherapeutic dressings on burn regeneration has been proven, as evidenced by changes of blood paramagnetic centers and free radicals, suggesting a pluripotent multifaceted influence of propolis contained in nonwovens on oxidative balance changes [42].

Summarizing the electrospinning process of the PLGA copolymer solution, regular, smooth fibers, arranged in a chaotic manner, without visible deformations and defects, have been obtained. The presence of bee product does not interfere with fiber formation during the electrospinning process. PLGA polymer fibers containing a larger amount of glycolidyl units in relation to lactidyl units showed a greater degree of degradation, which is caused by hydrophilic character of glycolic acid. The fastest propolis release was from PLGA 50/50 mats and the slowest from PLGA 85/15 mats, which is related to polymer degradation and indirectly to the effect of hydrophilic glycolic acid on the carrier properties. The release rate depended on the propolis content in the polymer matrix, which was particularly evident for the PLGA 50/50 samples. Modification of the copolymer unit ratio allows to control of the release profile of the drug substance from the mat. PLGA nonwovens with bee product give promising therapeutic effects. The best therapeutic effect was observed in treatments using PLGA with content 5% of bee product, which can be the most favorable concentration of the active compound to treat a wound.

The release of the drug is related to degradation of polymer carrier—the sooner the degradation occurs, the faster the drug will be released. The results showed that the fastest and largest amount of propolis was released from the PLGA 50/50 matrix. In turn, the slowest, and hence, the smallest amount of drug, was released from the PLGA 85/15 samples, which coincides with the degradation results. After 2 weeks of incubation, the difference in the drug release kinetics of particular types of nonwovens disappears, but the release occurs at constant and similar level. The difference in drug release kinetics between samples at the early stages of incubation is very important, because it allows to choose the appropriate release profile to saturate the damaged tissue. 

## 5. Conclusions

The nonwoven polymer obtained by electrospinning is potentially a promising dressing material characterized by much better properties than traditional dressings. Biodegradable, nonwoven dressings containing propolis release the apitherapeutic in a controlled manner.

## 6. Patents

Presented nonwovens and a method for the production of nonwovens are patent pending.

## Figures and Tables

**Table 1:** Properties of polymer material used for electrospinning of nonwovens for in vitro study.

Sample	Copolymer	M_n_ [kDa]	M_w_ [kDa]	D	T_g_ [°C]
PLGA 85/15	(l-LA 83%: GL 17%)	42.0	104.1	2.48	57
PLGA 70/30	(l-LA 68%: GL 32%)	48.9	102.0	2.1	53
PLGA 50/50	(l-LA 49%: GL 51%)	31.9	78.6	2.46	48

Copolymer–ratio of comonomers in copolymer; M_n_—number-average molar mass determined by gel permeation chromatography (GPC) and calibrated with polystyrene standards; M_w_—weight-average molar mass determined by GPC and calibrated with polystyrene standards; D—dispersity.

**Table 2:** Glass transition temperature (T_g_) [°C] of electrospun nonwovens.

Content of Propolis	None	5%	10%
Unit Ratio	T_g_ [°C]		
50/50	48	47	43
70/30	53	51	50
85/15	57	54	53

**Table 3:** Comonomeric unit content of different nonwovens during degradation obtained on the basis of ^1^H NMR spectra ($\bar{x},\;N=3$).

Content of Propolis		None		5%		10%	
Unit Ratio	Degradation Time [weeks]	[l-LA] [%]	[GL] [%]	[l-LA] [%]	[GL] [%]	[l-LA] [%]	[GL] [%]
50/50	0	49	51	49	51	49	51
	6	55	45	54	46	53	47
	12	60	40	57	43	57	43
70/30	0	68	32	68	32	68	32
	6	68	32	69	31	69	31
	12	71	29	71	29	71	29
85/15	0	83	17	83	17	83	17
	6	83	17	84	16	84	16
	12	83	17	84	16	84	16

[l-LA][%]–average content of lactidyl units, [GL][%]–average content of glycolidyl units.
